# Effects of Media Literacy Intervention on Weight-Control Products Digital Marketing Targeting Adolescents

**DOI:** 10.3390/bs14111023

**Published:** 2024-11-01

**Authors:** Li-Chuan Lin, Fong-Ching Chang, Tzu-Fu Huang, Tai-Yu Chen, Chiung-Hui Chiu, Ping-Hung Chen, Nae-Fang Miao, Hung-Yi Chuang, Hsueh-Chih Chen

**Affiliations:** 1 Department of Health Promotion and Health Education, National Taiwan Normal University, Taipei 10610, Taiwan; 80505002e@gapps.ntnu.edu.tw (L.-C.L.); 80705001e@ntnu.edu.tw (T.-F.H.); 60905004e@gapps.ntnu.edu.tw (T.-Y.C.); 2 Graduate Institute of Information and Computer Education, National Taiwan Normal University, Taipei 10610, Taiwan; cchui@ntnu.edu.tw; 3 Graduate Institute of Mass Communication, National Taiwan Normal University, Taipei 10610, Taiwan; pxc24@ntnu.edu.tw; 4 Post-Baccalaureate Program in Nursing, Taipei Medical University, Taipei 11031, Taiwan; naefang@tmu.edu.tw; 5 Department of Community Medicine, Kaohsiung Medical University Hospital, Kaohsiung 80708, Taiwan; ericch@kmu.edu.tw; 6 Department of Public Health, Kaohsiung Medical University, Kaohsiung 80708, Taiwan; 7 Department of Educational Psychology and Counseling, National Taiwan Normal University, Taipei 10610, Taiwan; chcjyh@ntnu.edu.tw

**Keywords:** adolescent, media literacy, weight-control product digital marketing, education intervention

## Abstract

This study aimed to evaluate the effects of a media literacy education intervention on adolescents’ responses to digital marketing of weight-control products, focusing on media literacy, persuasion resistance efficacy, and purchase intention. Using a quasi-experimental design, the study involved 326 11th-grade students from a municipal high school in Kaohsiung City, Taiwan, with 189 students in the intervention group and 137 in the comparison group. Conducted in 2023, the intervention group participated in baseline and follow-up assessments and attended four 50 min media literacy sessions, while the comparison group completed only baseline and follow-up assessments with standard instruction. The results indicated that the media literacy intervention had positive effects on adolescents’ conceptual, attitudinal, and critical media literacy, as well as their persuasion resistance efficacy in relation to digital marketing of weight-control products. However, no significant effect was observed on purchase intention. In conclusion, media literacy interventions can effectively enhance adolescents’ media literacy and their ability to resist persuasion.

## 1. Introduction

Adolescence is a critical period for personal and social identity formation [1], and much of this development is now influenced by social media [2]. Social media platforms have created a ‘perfect storm’ that exacerbates body image concerns among girls, leading to body dissatisfaction, depressive symptoms, and disordered eating behaviors [3]. For instance, TikTok inundates child and adolescent users with rapid-weight-loss videos, with tens of thousands of such videos appearing within weeks of joining the platform [4]. Studies have shown that adolescents are often unknowingly affected by biased social media algorithms, which worsen negative body image and contribute to eating disorders, mental health issues, and suicidality [5,6]. Weight-loss products are frequently adulterated with illegal and toxic ingredients, leading to serious side effects [7,8], including disordered eating behaviors [9], hepatotoxicity [10], cardiac complications, and even death [11]. Research has shown that the regulation of weight-loss supplements is weak in many countries, posing a significant threat to consumer health [12].

Body image is a crucial element of self-concept, particularly during adolescence when concerns about appearance become more prominent [13,14]. Among boys and girls, 44.7% and 40.3%, respectively, experienced moderate to clinically significant body dissatisfaction [15]. A review study found that social media use leads to body image concerns and eating disorders through the mediating pathways of social comparison, thin-ideal internalization, and self-objectification [16]. In some countries, such as the United States, social media influencers are required to disclose sponsorships to protect consumers from misleading practices. However, research has shown that combining sponsorship disclosure with media literacy interventions was more effective at activating consumers’ persuasion knowledge and reducing purchase intentions than disclosure alone [17].

Children and adolescents often struggle to resist digital marketing, prompting experts to advocate for the development and implementation of digital health and media literacy programs to enhance their critical-thinking skills [18,19,20]. One study found that media literacy, particularly critical thinking, can mitigate the negative effects of thin-ideal internalization and upward appearance comparisons on body satisfaction [21]. Canada’s Centre for Digital Media Literacy emphasizes that digital media literacy involves the ability to think critically and engage responsibly with digital media [22]. Scholars have proposed two dimensions of advertising literacy: conceptual, which refers to the ability to recognize and understand advertising messages, and attitudinal, which involves skepticism and a critical attitude toward advertising [23,24]. Media literacy education helps young people actively evaluate and analyze media content [25,26]. School-based interventions have been shown to improve media literacy and reduce body dissatisfaction and eating concerns [27,28]. For example, a social media literacy intervention for adolescent girls improved media literacy, body image, and disordered eating behaviors [29]. Additionally, media literacy education interventions positively impacted dietary restraint and reduced depressive symptoms in adolescent girls [30].

In Taiwan, approximately 30% of individuals under the age of 18 are classified as overweight or obese, while over 10% are underweight. These rates have been increasing over the past decade. Meanwhile, social media use among adolescents reached 96% in 2023. On these platforms, companies often employ influencers to promote weight-control products. However, Taiwan currently lacks regulations regarding influencer sponsorship disclosure, raising concerns among experts and parents that children and adolescents are particularly susceptible to influencer marketing. While studies [31,32] have established a link between media exposure and body dissatisfaction, and research [27] suggests that media literacy can improve adolescents’ body image, there remains a significant gap in research on educational interventions addressing the digital marketing of weight-control products in Asian societies. Few studies have developed digital media marketing literacy programs or examined their effects on adolescents’ ability to resist influence and their purchasing behaviors. This study aims to address this gap by integrating the Persuasion Knowledge Model with resistance theories to develop and evaluate a media literacy education intervention focused on the digital marketing of weight-control products in a school setting. The study examined the intervention’s impact on high school students’ media literacy, persuasion resistance efficacy, and purchase intentions.

## 2. Materials and Methods

### 2.1. Study Design and Population

This study utilized a quasi-experimental design. The intervention group completed both baseline and follow-up questionnaires and participated in an educational intervention, while the comparison group received study information and completed only the baseline and follow-up questionnaires. After obtaining informed consent, the baseline assessment was administered. One week later, the intervention group engaged in a media literacy education curriculum focused on weight-control products, which consisted of four 50 min sessions. A follow-up assessment was conducted one week after the course to evaluate the intervention’s impact on media literacy regarding the digital marketing of weight-control products.

The study, conducted from March to June 2023, involved participants from eleven 11th-grade classes at a high school in Kaohsiung City. Six classes were randomly assigned to the intervention group, and five to the comparison group. The initial sample included 378 students, but 29 from the intervention group and 23 from the comparison group were excluded due to absences or incomplete questionnaires. Ultimately, 326 students completed both test questionnaires—189 in the intervention group and 137 in the comparison group—yielding an overall response rate of approximately 86%.

### 2.2. Media Literacy Education Intervention

The media literacy education intervention for high school students was based on the Persuasion Knowledge Model (PKM) and resistance theories. This program was designed to empower students to develop critical-thinking skills and strengthen their resistance to mitigate the negative effects of digital marketing, enabling them to make informed health decisions. A review study suggested that empowering younger audiences and teaching them analytical skills can enhance the effectiveness of media literacy interventions [33]. Additionally, effective interventions should involve active participation, inquiry-based activities, and co-creation from both teachers and students, engaging them in tasks that foster critical-thinking skills and enhance digital media literacy [34,35,36].

In this study, the media literacy education intervention aimed to enhance students’ media literacy and resistance to persuasive messages regarding the digital marketing of weight-control products. The program included four key components: (1) Unmasking the Tricks: Students learn to recognize the purposes and tactics of digital marketing for weight-control products. (2) Digital Sleuths: Students explore the intricacies of digital marketing, including algorithms and sponsorship economic models. (3) Armor Up: Students practice resistance strategies against the digital marketing of weight-control products. (4) Creative Crusaders: Students create reverse marketing posts related to weight-control products, specifically tailored to adolescents. Videos and images were used as discussion materials to enhance student engagement and understanding.

### 2.3. Measurements

Questionnaires were used in this study to assess adolescents’ responses to digital marketing of weight-control products, focusing on media literacy, persuasion resistance efficacy, and purchase intention. Initially, a structured questionnaire draft was developed based on previous studies [21,23,24,37]. A panel of five experts—three professors specializing in information and computer education, mass communication, and health education, and two high school teachers—was invited to evaluate the questionnaire’s content validity. After discussions with the experts, the item descriptions were revised, and inappropriate items were removed based on their feedback. A pilot test was then conducted, followed by a reliability analysis using internal consistency measures. This process resulted in the final version of the questionnaire, which was used to evaluate the effects of the study. The questionnaire included the following components:

#### 2.3.1. Conceptual Media Literacy

Conceptual media literacy was adapted from the Persuasion Knowledge Scales of Sponsored Content (PKS-SC) and the children’s advertising literacy scale [23,24]. It included seven dimensions: recognition of advertising (2 items), recognition of sponsored content (3 items), understanding of selling intent (3 items), authenticity recognition of content (5 items), understanding of persuasive tactics (3 items), recognition of target audience (2 items), and knowledge of weight control (4 items). Sample statements included “I believe this post is sponsored by an advertiser” and “Advertisers embed products in posts to conceal commercial intent and reduce viewers’ skepticism”. Responses were rated on a four-point Likert scale: ‘Strongly agree’ (4 points), ‘Agree’ (3 points), ‘Disagree’ (2 points), and ‘Strongly disagree’ (1 point). Higher scores indicate a higher level of conceptual media literacy. Cronbach’s alpha for conceptual media literacy was 0.93.

#### 2.3.2. Attitudinal Media Literacy

Attitudinal media literacy was adapted from the Persuasion Knowledge Scales of Sponsored Content (PKS-SC) and the children’s advertising literacy scale [23,24]. It included two dimensions: skepticism toward posts (4 items) and disliking of posts (4 items). Sample statements included “It is dishonest when social media posts/videos about weight-loss products do not disclose sponsorship by the company” and “Seeing digital marketing advertisements for weight-control products on social media platforms is troubling”. Responses were rated on a four-point Likert scale: ‘Strongly agree’ (4 points), ‘Agree’ (3 points), ‘Disagree’ (2 points), and ‘Strongly disagree’ (1 point). Higher scores indicate a higher level of attitudinal media literacy. Cronbach’s alpha for attitudinal media literacy was 0.85.

#### 2.3.3. Critical Media Literacy

Critical media literacy was adapted from the Critical Thinking about Media Messages Appearance Focus (CTMM-AF) scale [21] and measured using six items. Sample statements included “When I see attractive models in weight-loss product advertisements, I understand that advertisers are trying to capture my attention”, and “When I see advertisements with slim or muscular models, I consider the authenticity of these weight-loss product advertisements”. Responses were rated on a four-point Likert scale: ‘Strongly agree’ (4 points), ‘Agree’ (3 points), ‘Disagree’ (2 points), and ‘Strongly disagree’ (1 point). Higher scores indicated a higher level of critical media literacy. Cronbach’s alpha for critical media literacy was 0.73.

#### 2.3.4. Persuasion Resistance Efficacy

Persuasion resistance efficacy was based on the comprehensive framework of persuasion resistance [37] and included four dimensions: avoidance strategies (7 items), contesting strategies (7 items), empowerment strategies (6 items), and biased processing strategies (3 items). Sample statements included “When I see advertisements for weight-loss products, I can deliberately avoid clicking on the page”, and “When faced with marketing messages for weight-loss products, I can identify the strategies being used to reduce their impact”. Responses were rated on a five-point Likert scale: ‘Very confident 100%’ (5 points), ‘Confident 75%’ (4 points), ‘Somewhat confident 50%’ (3 points), ‘Not confident 25%’ (2 points), and ‘Not confident at all 0%’ (1 point). Higher scores indicated greater confidence in resisting digital marketing persuasion. Cronbach’s alpha for persuasion resistance efficacy was 0.89.

#### 2.3.5. Purchase Intention

Purchase intention was measured using six items. Sample statements included “I am likely to purchase weight-loss products advertised on social media” and “I am likely to purchase weight-loss products recommended by influencers or peers”. Responses were rated on a four-point Likert scale: ‘Very likely’ (4 points), ‘Likely’ (3 points), ‘Unlikely’ (2 points), and ‘Very unlikely’ (1 point). Higher scores indicate a greater intention to purchase weight-loss products online. Cronbach’s alpha for purchase intention was 0.86.

#### 2.3.6. Demographic Variables

Demographic information collected included gender (male vs. female), Body Mass Index (BMI), weight perception (slightly underweight, about the right weight, or slightly overweight), and body image (dissatisfied vs. satisfied). Participants provided their height and weight, from which BMI values were calculated and classified as underweight, normal, overweight, or obese according to the Ministry of Health and Welfare’s guidelines for children and adolescents.

### 2.4. Statistical Analysis

Statistical analyses were conducted using the SAS 9.4 software package. Categorical data were described with frequencies and percentages, while interval data were summarized using means and standard deviations. Chi-square tests (χ^2^) were employed to assess differences in frequency distribution between the intervention and comparison groups. Paired *t*-tests were conducted to compare baseline and follow-up differences in the indicators for both groups. Generalized Estimating Equations (GEEs) were used for inferential statistics to evaluate the effects of the media literacy education intervention on participants’ digital marketing literacy, persuasion resistance efficacy, and weight-control product purchase intentions. The null hypothesis was tested at a significance level of α = 0.05.

## 3. Results

### 3.1. Socio-Demographic Characteristics

Table 1 presents the socio-demographic characteristics of students in the intervention and comparison groups. In the intervention group, 59.3% of the students were male, and 40.7% were female. Chi-square test results revealed a significant difference in gender distribution between the groups. At baseline, no significant differences were found between the intervention and comparison groups regarding BMI, perceived weight, and body image satisfaction, indicating comparability between the groups.

Baseline data indicated that approximately 25.9% of students had a BMI classified as overweight or obese, while 10.8% were classified as underweight. Additionally, 42.6% of students perceived themselves as overweight, and 14.1% perceived themselves as underweight. Further analysis revealed that among students with a normal BMI, 37.5% perceived themselves as overweight, and 6.8% perceived themselves as underweight. These results suggest a tendency for weight misperception among participants. Furthermore, about one-third of the students reported dissatisfaction with their body image (Table 1).   

### 3.2. Changes in Media Literacy, Persuasion Resistance Efficacy, and Purchase Intentions

Table 2 presents the changes in students’ media literacy, persuasion resistance efficacy, and purchase intentions from baseline to follow-up between the intervention and comparison groups. In the intervention group, the mean score for conceptual media literacy increased from 3.18 at baseline to 3.29 at follow-up. Similarly, critical media literacy scores rose from 3.04 at baseline to 3.28 at follow-up. Additionally, the mean score for persuasion resistance efficacy increased from 3.77 at baseline to 3.98 at follow-up. Paired *t*-tests indicated significant improvements in the intervention group for conceptual media literacy, critical media literacy, and persuasion resistance efficacy from baseline to follow-up, while no significant changes were observed in the comparison group across all indicators. Within the domain of persuasion resistance efficacy, students scored highest on “I can refrain from commenting or responding to marketing posts” and lowest on “I can use ad-blocking applications”. Furthermore, the mean score for purchase intentions in the intervention group slightly decreased from 1.89 at baseline to 1.85 at follow-up.

### 3.3. Intervention Effects on Media Literacy

Table 3 presents the effects of the media literacy educational intervention on conceptual, attitudinal, and critical media literacy regarding digital marketing of weight-control products. Generalized Estimating Equation (GEE) analysis revealed that, after controlling for gender, the intervention had significant positive effects on students’ conceptual media literacy (β = 0.09, *p* < 0.05), attitudinal media literacy (β = 0.14, *p* < 0.05), and critical media literacy (β = 0.21, *p* < 0.05). Furthermore, after controlling for gender, BMI, weight perception, and body dissatisfaction, the GEE analysis indicated that the intervention continued to show positive effects on students’ conceptual, attitudinal, and critical media literacy.

### 3.4. Intervention Effects on Persuasion Resistance Efficacy and Purchase Intentions

Table 4 presents the effects of the media literacy educational intervention on persuasion resistance efficacy and purchase intentions regarding digital marketing of weight-control products. GEE analysis revealed that, after controlling for gender, the intervention had significant positive effects on students’ persuasion resistance efficacy (β = 0.22, *p* < 0.05). However, the intervention showed no significant effect on students’ purchase intentions (β = −0.07, *p* = 0.35), although a trend toward decreased purchase intentions was observed in the intervention group. Furthermore, after controlling for gender, BMI, weight perception, and body dissatisfaction, the GEE analysis indicated that the intervention continued to show positive effects on students’ persuasion resistance efficacy.

## 4. Discussion

The results showed that media literacy interventions effectively improved adolescents’ conceptual, attitudinal, and critical media literacy, fostering greater skepticism toward digital marketing of weight-control products. Previous studies have demonstrated that school-based media literacy programs can enhance media literacy and reduce body dissatisfaction and eating concerns among adolescents [27,29,30]. These findings highlight the importance of integrating media literacy into health education to enhance adolescents’ media literacy skills. Schools should also educate adolescents about the dangers of weight-loss products and promote a positive body image [38]. Additionally, parental mediation plays a crucial role in shaping adolescents’ media literacy and purchasing behaviors. Research suggests that media literacy interventions should adopt a family-centered approach, emphasizing family communication to empower both parents and adolescents. This strategy fosters critical discussions, reduces the influence of digital marketing, and helps prevent obesity and eating disorders [39,40]. Health professionals should also support parents and adolescents in developing digital literacy skills to critically evaluate digital marketing tactics [20].

Our findings demonstrated that the media literacy educational intervention effectively improved adolescents’ ability to resist digital marketing for weight-control products. A prior study showed that adolescents’ advertising literacy positively influences their resistance to advertising [41]. Studies integrating resistance theories with the Persuasion Knowledge Model (PKM) found that disclosing online native advertising activates persuasion knowledge, enabling adolescents to employ cognitive and affective resistance strategies to counter persuasive attempts [42]. In addition, research has indicated that disclosing sponsored blogs, combined with media literacy education, could enhance adolescents’ resistance to persuasion [17]. These studies provided empirical support for the concept that consumers use resistance strategies when their persuasion knowledge is activated. Our findings also highlighted that learning to use tools such as ad blockers and incognito mode improved adolescents’ ability to resist digital marketing. Furthermore, the World Health Organization has advocated for government regulation of digital marketing to protect children and adolescents from exposure to unhealthy products [43].

This study found that the intervention had no significant impact on purchase intentions, although a trend toward decreased purchase intentions was observed in the intervention group. This may be due to the adolescents’ initially low intention to purchase weight-control products, which made the decrease less statistically significant. In addition, the study revealed that among adolescents with a normal BMI, over one-third perceived themselves as overweight, while 6.8% considered themselves underweight, highlighting a significant issue of weight misperception. Another study similarly found that more than a fifth of adolescents misperceive their weight [44]. These misperceptions can influence dietary behaviors and physical activity, potentially increasing the risk of eating disorders and obesity [45,46,47]. Accurate weight perception is critical for health, as it strongly predicts dietary habits, physical activity, and effective weight management [48,49]. Therefore, healthcare providers, public health professionals, and educators should develop interventions to educate adolescents and parents about proper body weight, healthy weight management, exercise, and nutrition [44].

This study had several limitations. First, adolescents’ self-reported height and weight may have introduced recall bias, suggesting that future studies should directly measure these metrics to improve validity. Second, the evaluation included only a single follow-up assessment, limiting the ability to examine the long-term effects of the intervention. Lastly, due to the structure of the sample school, with eleven 10th-grade classes, equal sample sizes for the intervention and comparison groups could not be achieved. Classes were randomly assigned, resulting in six classes in the intervention group and five in the comparison group, leading to a larger number of students in the intervention group. Despite these limitations, this study developed a media literacy educational intervention focused on the digital marketing of weight-control products and evaluated its effects on adolescents’ media literacy, persuasion resistance efficacy, and purchasing intentions. This intervention could serve as an educational model for future research in this area.

## 5. Conclusions

This study demonstrated that the media literacy educational intervention had positive effects on enhancing adolescents’ conceptual, attitudinal, and critical media literacy, and resistance to digital marketing of weight-control products. Adolescents improved their ability to understand and apply critical skills to resist the influence of digital marketing. Activating persuasion knowledge, as outlined in persuasion theory, empowers consumers to recognize and respond to persuasive attempts by effectively employing resistance strategies, providing empirical support for this approach. Future research suggests integrating media literacy into school health programs to further strengthen adolescents’ critical thinking and resistance to unhealthy digital products. Studies also emphasize the importance of addressing diverse digital marketing formats and incorporating inquiry-based and co-creation activities to support ongoing media literacy development, ultimately improving adolescents’ digital health and media literacy [35,36].

## Figures and Tables

**Table 1:** Demographic characteristics in the intervention and comparison groups.

Variables	Total		Intervention Group		Comparison Group		*p*-Value
	n	%	n	%	n	%	
Gender							<0.001
Female	166	50.9	77	40.7	89	65.0	
Male	160	49.1	112	59.3	48	35.0	
Body Mass Index							0.792
Underweight	35	10.8	19	10.1	16	11.7	
Normal	206	63.4	122	64.9	84	61.3	
Overweight/obesity	84	25.9	47	25.0	37	27.0	
Weight Perception							0.882
Slightly underweight	46	14.1	28	14.8	18	13.1	
About the right weight	141	43.3	80	42.3	61	44.5	
Slightly overweight	139	42.6	81	42.9	58	42.3	
Body Image							0.205
Dissatisfied	111	34.1	59	31.2	52	38.0	
Satisfied	215	66.0	130	68.8	85	62.0	

Chi-square tests were conducted.

**Table 2:** Changes in media literacy, persuasion resistance efficacy, and purchase intentions.

Variables (No. of Items)	Intervention Group		Comparison Group			
	Baseline (n = 189)	Follow-Up (n = 189)	*p*-Value	Baseline (n = 137)	Follow-Up (n = 137)	*p*-Value
	Mean (SD)	Mean (SD)		Mean (SD)	Mean (SD)	
Conceptual media literacy (22)	3.18 (0.38)	3.29 (0.53)	0.001	3.23 (0.36)	3.26 (0.45)	0.464
Attitudinal media literacy (8)	3.33 (0.45)	3.39 (0.59)	0.117	3.39 (0.48)	3.30 (0.53)	0.069
Critical media literacy (6)	3.04 (0.53)	3.28 (0.74)	<0.001	3.13 (0.49)	3.16 (0.53)	0.407
Persuasion resistance efficacy (23)	3.77 (0.61)	3.98 (0.71)	<0.001	3.77 (0.66)	3.75 (0.76)	0.584
Purchase intention (6)	1.89 (0.47)	1.85 (0.49)	0.262	1.93 (0.52)	1.95 (0.57)	0.734

Paired *t*-tests were conducted.

**Table 3:** The media literacy intervention’s effects on media literacy.

Variables	β	SE	*p*-Value
Conceptual media literacy			
Intercept	3.27	0.04	<0.001
Pre- and post-test	0.02	0.03	0.404
Group	−0.03	0.04	0.547
Test × Group	0.09	0.04	0.040
Gender	−0.10	0.04	0.016
Attitudinal media literacy			
Intercept	3.46	0.05	<0.001
Pre- and post-test	−0.08	0.04	0.061
Group	−0.01	0.05	0.827
Test × Group	0.14	0.06	0.018
Gender	−0.17	0.05	0.000
Critical media literacy			
Intercept	3.13	0.06	<0.001
Pre- and post-test	0.04	0.04	0.358
Group	−0.07	0.06	0.260
Test × Group	0.21	0.07	0.005
Gender	−0.07	0.06	0.209

N = 326 students, observation n = 652. The GENMOD procedure with the REPEATED statement was used to estimate the coefficients. Y = β_0_ + β_1_ (test) + β_2_ (group) + β_3_ (test × group) + β_4_ (gender); Y: conceptual media literacy, attitudinal media literacy, and critical media literacy score; test: follow-up test = 1, baseline test = 0; group: intervention group = 1, comparison group = 0; gender: male = 1, female = 0.

**Table 4:** The media literacy intervention’s effects on persuasion resistance efficacy and purchase intentions.

Variables	β	SE	*p*-Value
Persuasion resistance efficacy			
Intercept	3.83	0.07	<0.001
Test	−0.03	0.05	0.570
Group	0.02	0.07	0.735
Test × Group	0.22	0.07	0.001
Gender	−0.07	0.07	0.346
Purchase intention			
Intercept	1.92	0.05	<0.001
Test	0.03	0.06	0.647
Group	−0.01	0.06	0.853
Test × Group	−0.07	0.07	0.360
Gender	−0.08	0.05	0.062

N = 326 students, observation n = 652. The GENMOD procedure with the REPEATED statement was used to estimate the coefficients. Y = β_0_ + β_1_ (test) + β_2_ (group) + β_3_ (test × group) + β_4_ (gender); Y: persuasion resistance efficacy and purchase intention score; test: follow-up test = 1, baseline test = 0; group: intervention group = 1, comparison group = 0; gender: male = 1, female = 0.

## Data Availability

The data presented in this study can be requested and provided.
